# Influence of personality traits on generation Z consumers' click-through intentions towards personalized advertisements: A mixed-methods study

**DOI:** 10.1016/j.heliyon.2024.e34559

**Published:** 2024-07-17

**Authors:** Partha Saha, Angan Sengupta, Priya Gupta

**Affiliations:** Amrita School of Business, Amrita Vishwa Vidyapeetham, Bengaluru, India

**Keywords:** Social media advertising, Mixed methods, Big-five personality traits, Perceived personalization, Privacy paradox

## Abstract

Personalized social media advertisements (PSMAs) are developed by using consumers' personal information like names, demographic details, location, past buying history, and lifestyle interests to quickly grab consumers' attention within the cluttered space of digital advertisements. Generation Z consumers are highly connected to social media. Hence, this study attempts to understand how Generation Z consumers with different personality traits perceive personalized advertisements (PAs) on Facebook, and subsequently, how their perceived personalization influences their intention to click on PAs based on perceived usefulness and privacy concerns associated with those advertisements. The theoretical underpinning of this research is based on the self-congruency theory and privacy-calculus theory. An explanatory sequential mixed-methods design has been adopted, where a quantitative analysis (Study 1) is followed by a qualitative approach (Study 2). For Study 1, responses were collected from 324 Generation Z consumers through a structured questionnaire and the data was analyzed using the structural equation modeling technique to measure relationships among the constructs. Further, in Study 2, in-depth interviews were conducted with 15 Generation Z consumers, a purposively selected subset of informants from Study 1, to explore the potential causes of those relationships observed in Study 1. It has been found from the study that consumers' perception of PSMAs varies based on their personality traits. Consumers with dominant extraversion, conscientiousness, and neurotic personality traits perceive PSMAs positively whereas the openness to experience and agreeableness dominant consumers perceive those negatively. Positive perception of PSMAs among consumers increases the perceived usefulness of communications and subsequently improves the click-through intentions of the consumers. Generation Z Consumers' perception of PSMAs does not have any influence on their privacy concerns. Consumers’ high privacy concerns reduce the click-through intention rate toward PSMAs. The study will help digital marketing managers to strategically deliver PSMAs, thereby enhancing the efficiency of their advertisements.

## 1 Introduction

The advertisement revenue of Meta, formerly known as Facebook, increased 23 % from USD 113.64 billion in 2022 to USD 131.94 billion in 2023 [1]. This staggering growth is fueled by the irresistible power of personalized advertising (PAs), where the company utilizes consumers' personal information like names, demographics, geographical details, past buying habits, and lifestyle preferences for crafting marketing communications [2]. Facebook is considered one of the best social media platforms for the distribution of PAs [[3], [4], [5], [6]]. It uses machine-learning models on consumers' previous behavior data to decide the most suitable trait-based personalized social media advertisements (PSMAs) for every user [7]. PAs can be categorized into cue-based PAs and trait-based PAs based on the personalization techniques and elements. Cue-based PAs use individuals' identifiable information. In trait-based PAs, messages are customized based on individual consumer's needs and interests [2,8].

Researchers found that along with informativeness, credibility, and entertainment; personalization is also an important attribute of advertisements that has incredible value in creating positive attitudes among consumers toward the brands [3,9]. Personalization helps brands to create one-to-one relationships with customers [10]. PAs reduce consumers' efforts of searching on digital mediums by communicating the right advertisement to the recipient at the right time [11]. The success of trait-based PAs depends not only on the personalization strategies used for crafting the messages but also on whether the recipient perceives the messages as customized based on her/his needs [12]. Consumers' perceived personalization (PP) is an important determinant that decides the performance of any PAs. Self-congruity of individuals creates the recipient's perception toward communication [[13], [14], [15]]. The influence of trait-based PAs differs on consumers for several reasons like, consumers' cultural differences [16], age differences, or even differences in consumers' psychological determinants like personality traits [17].

Statistics suggest that more than 23 % of Facebook users belong to the 18–24 years age group. This age group population forms a significant size of the Generation Z population category (who were born after 1997) [18]. Therefore, it will be interesting to understand the perception of PA among the Generation Z population [19]. Understanding their attitudes toward personalization is crucial for the advancement of the field, given their widespread presence as internet users and e-commerce customers [20,21]. Generation Z consumers are heavily exposed to digital communications and ready to share their personal data with brands that offer tailored experiences [22]. However, they often become frustrated and employ avoidance tactics when brands fail to meet their unique needs [23]. According to the self-congruity theory, if any message fits with a consumer's dominant personality trait, then that increases the consumer's interest in that message [13,24], improves the perceived relevance of social media ads, and subsequently reduces the chance of ad avoidance [25]. The research also found that the intentions of purchasing products after observing PAs significantly differ among different personality traits individuals [17]. Hence, there is a need for empirical examination attempting to understand the varying interests of Generation Z consumers with different personality traits on the trait-based PSMAs. In this work, the self-congruity theory has been considered as the base theory for examining the relationships among consumers' dominant personality traits and PP. This study employed Costa and McCrae's Big Five personality trait framework, which classifies individuals into five major traits: extraversion, agreeableness, conscientiousness, neuroticism, and openness to experience [26].

Though PAs raise interest among recipients, people still hesitate to interact with trait-based PAs because of privacy issues. They fear losing personal information interacting with those PAs [27,28]. This phenomenon is popularly known as the ‘Personalization-Privacy paradox’. According to the privacy calculus theory (PCT), individuals often compare the perceived benefits and risks before disclosing personal information in any context [29]. Underpinning the privacy calculus theory, Mckee and team [29]empirically proved that Generation Z consumers' perceived benefits toward a company's personalized marketing efforts increase loyalty toward the brand whereas privacy concerns decrease the association with the brand. Different research works also examined the trade-off between consumers' perceived usefulness (PU) and privacy concerns (PC) on the click-through intentions of PSMAs [23,30]. However, no study investigated whether consumers' PP has any influence on the determinants of the privacy-benefit paradox, i.e. PU and PC. In this study, researchers further tried to expand the privacy-calculus theory by incorporating the PP construct. The extant literature could identify the need for examining how consumers' PP influences PU and PC and subsequently influences consumers' click-through intentions on the trait-based PAs.

Hence, the primary objective of this work is to examine whether the click-through intentions toward PSMAs among Generation Z consumers with different personality traits are varied or similar, considering the influence of PP and the personalization-privacy paradox. Furthermore, we seek to explore the reasons and rationale of consumers from each dominant personality trait that shapes their perceived personalization towards PSMAs and subsequently influences the personalization-privacy paradox for engaging with those advertisements. Three research questions have been developed to achieve the primary research objective.

RQ1 Does the perception of Generation Z consumers toward trait-based personalized social media advertisements (PSMAs) vary according to the personality traits of the consumers?

RQ2 Do Generation Z consumers' perceptions toward trait-based personalized social media advertisements (PSMAs) influence consumers' perceived usefulness and privacy concerns toward the advertisements, and subsequently impact their click-through intentions toward those advertisements?

RQ3 What are the factors that influence consumers' perceived personalization, perceived usefulness, and privacy concerns towards trait-based PSMAs?

## 2 Literature review

### 2.1 Consumers’ personality traits and personalized advertisements

Research works confirmed that persons with different personality traits react to communications differently [[31], [32], [33]]. Extroverted people prefer to be with others, go out with friends, and like to socialize. These types of persons showed favorable attitude toward both physical and online advertisements which becomes a source of added information for them and fulfills their need for cognition. It was also observed that introverted people didn't show any significant behavior toward advertisements [34]. People with dominantly extroverted and open-to-experience traits usually like to share informational advertisements more than emotional advertisements, since they are rich in information and capable to influence people [35]. Another study also showed that extroverted people prefer informational advertisements over that of promotion-focused ones [25].

Studies have also noticed that the impact of Personalized Advertisements (PAs) varies with the psychological traits of the recipients [17]. The effectiveness of the advertisement increases if the message frame is congruent with the recipient's personality trait. Message framing based on personality traits improves the perceived relevance of social media ads. Subsequently, it reduces the chance of ad avoidance which supports the self-congruency theory [25]. Consumers with high scores in extraversion prefer content with excitement, achievements, and social recognition; while people with high agreeableness tend to like family and community bonding. Meanwhile, consumers with high conscientiousness love to watch preciseness, accuracy, and efficiency. Consumers with high neuroticism are eager about safety and security whereas consumers with high scores in openness to experience look for innovation and creativity [36].

**Self-congruity theory:** Self-congruity theory posits that people act more favorably with people or messages that are aligned with their perceptions and beliefs [24,[37], [38], [39]]. That also creates a positive attitude toward the advertisement [40] and subsequently toward the brand [38]. Similarly, self-congruent advertisements show comparatively better effectiveness than non-congruent messages in advertisements [41]. Hong and Zinkhan [24]observed in their experiment that extroverted-appealed and introverted-appealed advertisements separately influenced trait-dominant consumers' purchase intentions and brand preferences more than non-congruent advertisements. Research showed that self-congruency has significant relationships with all the big five personality traits, proposed by Costa and McCrea [42]. Research has also shown that messages framed based on personality traits improve the perceived relevance of social media ads and subsequently reduce the chance of ad avoidance [5,17,25]. In this research work, the theory of self-congruency has been extended by examining the relationships between consumers’ personality traits and PP.

**Big-five personality traits:** Personality is one of the important psychological determinants of human behavior. Costa and McCrea's Big-five personality trait framework has been considered for this study. Costa and McCrae started to develop the Big-Five Inventory scale around 1980. Eventually, in 1992, they developed a 240-item NEO Personality Inventory [26]. Further modifying the NEO Personality Inventory, a 44-item Big Five Inventory (BFI) was constructed [43]. These Big-five personality traits are extraversion, agreeableness, conscientiousness, neuroticism, and openness to experience [26]. People with extraversion personality dimension prefer to be surrounded by people and have a talkative, energetic, and adventurous nature [26]. They aspire to hold leadership positions and take risks. Agreeable people are empathetic, compassionate, and generous by nature [26]. These types of people prefer to trust others and always agree to help others. People feel warmth in the presence of them. Without the presence of this characteristic, people are considered rude, cruel, and self-centered. People who score high on conscientiousness are naturally disciplined [26]. They try to adhere to all the rules and regulations and take responsibility and ownership of tasks. People can easily rely on this kind of person for the completion or execution of tasks or jobs. Costa and McCrae [26] mentioned about people with neuroticism as those who cannot control their emotions or impulses and get easily agitated in any given situation(s). Another personality trait that is crucial to understand behavior is openness to experience. People who score high on this trait are very curious about new complex situations, are open-minded, always ready to learn and accept new things and ideas, forward-thinking, imaginative, and display special artistic senses [26]. The Big-Five Personality Trait scale has been utilized in several earlier studies to understand human characteristics and behaviors [25,[44], [45], [46]].

**Personality traits and perceived personalization:** PAs are advertisements where some personal element(s) of the recipient is incorporated with the communication to enhance receiver's interest and grab their attention [47]. However, advertisements though created by using personalization strategies, sometimes fail to evoke self-association with the consumers [2]. Therefore, sometimes even PAs have been considered generic ads by consumers [48]. Consumers must appraise that the advertisements have been uniquely designed for her/him [49]. Trang Tran has found out that consumers' perceptions of personalization motivate consumers to act on the ads [4]. Rather than actual personalization, perceived personalization (PP) makes consumers more aware that the message has been developed for the recipient specifically. Ewa Maslowska and team are the first who experimented and confirmed that PP acts as a mediator to increase the engagement of consumers with PAs which also supports the self-congruity theory [49]. Cong Li also emphasized in his work that rather than actual personalization, consumers' PP defines personalized communications effectiveness [12]. The concept of PP is not only studied in the advertisement domain but also in other domains like hotel & tourism, healthcare, etc. [[50], [51], [52]]. Out of different personalization elements, consumers' interests, location, and age, are more effective to induce PP among the recipients [53].

Researchers have studied the importance of consumers' PP on the success of personalized campaigns [5,17]. However, there is no prominent study that examined how consumers with different dominant personality traits perceive personalized social media advertisements (PSMA). In this study, the first research objective is to measure whether consumers' PP toward the PSMAs remains the same or varies based on the consumer's dominant personality trait. It is also crucial to understand how consumers with different personality traits perceive PSMAs. Since PAs may lead to annoyance among consumers rather than creating positive interest in them, that can be detrimental to the brand [54]. For this study, the following hypotheses have been formulated.

H1a Generation Z consumers with high scores in the Extraversion trait perceive PSMAs positively.

H1b Generation Z consumers with high scores in the Agreeableness trait perceive PSMAs negatively.

H1c Generation Z consumers with high scores in the Conscientiousness trait perceive PSMAs positively.

H1d Generation Z consumers with high Neuroticism traits perceive PSMAs positively.

H1e Generation Z consumers with high scores in the Openness trait perceive PSMAs negatively.

### 2.2 Personalization-privacy paradox

**Privacy calculus theory:** Laufer and Wolfe [55] first proposed the concept of privacy calculus. According to the privacy-calculus theory (PCT), individuals often compare the perceived benefits and risks before disclosing personal information in any particular context. It is the function of the consumer's positive and negative perceptions that decide how much information to disclose and where to disclose [56]. The theory was first applied in the field of information systems [57] and was later applied in the areas of adoption of mobile applications [58], making relationships with unknown vendors in e-commerce [59], and studying people's decision-making processes using digital health platforms [[60], [61], [62]]. Researchers also used PCT as the base theory to understand the personalization-privacy paradox related to personalized advertisements. Anabel Gutierrez and colleagues used this theory in their scientific work on consumers' adoption of mobile location-based advertising [63]. Gironda and Korgaonkar [27] researched the consumers' perceptions of PAs keeping the PCT as the underpinning theory of the work. The theory was extended with other constructs like invasiveness and consumer innovativeness. Another study found that a strong consumer-brand relationship improves the perceived benefits of disclosing personal information for PAs whereas it reduces privacy concerns [64]. In this study, the PCT theory has been extended by examining the influence of consumers' PP on their perceived usefulness (PU) and privacy concerns (PC). Subsequently, the study has also examined the influence on consumers' click-through intentions.

**Perceived personalization & perceived usefulness:** Zahay and colleagues [65] showed that the effectiveness of PAs depends on the consumers' perceptions of the utility of the messages. Perceived Usefulness (PU) helps consumers to perceive that the message will improve their job performances [66]. A few studies have been conducted to explore the antecedents of perceived usefulness related to the PAs. Research showed that knowledge of personalization of the advertisements improves consumers' perception of the benefits and value of those [64,67]. Even consumers' knowledge about persuasion strategies also has a positive impact on the PU [68]. Research also showed that consumers' PP improves the relevance of online and social media ads [69,70] but as far as our knowledge goes, no study examined the influence of Generation Z consumers' PP on their PU toward the PAs. Keeping that research gap in mind, this study has examined whether Generation Z consumers' PP influences consumers’ PU of the PSMAs or not. The hypothesis developed for the research is mentioned below.

H2 Generation Z consumers' high perceived personalization towards PSMAs increases their perceived usefulness of those ads.

**Perceived personalization & privacy concerns:** Burgoon has defined privacy as “the ability to control and limit physical, interactional, psychological, and informational access to the self or one's group” [71]. Privacy concern (PC) is a construct developed to understand the uneasiness people feel due to the usage of their personal information by different organizations [72,73]. Since PAs are developed using consumers' personal information, consumers feel that they have less control over their personal information, which in turn increases consumers' privacy concerns [27]. It has been observed that trust in the organization helped to reduce consumers' privacy concerns toward that organization's PAs [30]. Proper disclosure of the personalization process also reduces consumers' fear of information theft [74]. A study conducted on web personalization showed that the higher a consumer's PC lower be consumer's trust or web loyalty [75]. Consumers are also concerned about the overt and covert data collection techniques used by companies to collect consumers' personal information [2]. In this study, the influence of consumers' perceived personalization on privacy concerns has been researched. The hypothesis formulated is mentioned below.

H3 Generation Z consumers' high perceived personalization toward PSMAs reduces their privacy concerns about those ads.

**Fig. 1:**
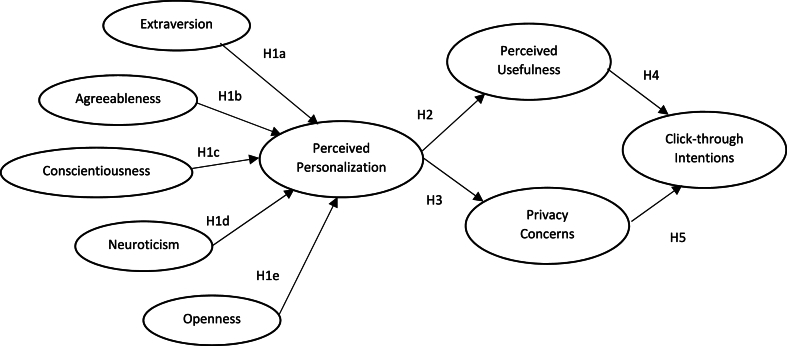
Conceptual framework diagram.

**Personalization-privacy paradox & click-through intentions:** Research works have observed that consumers' perceived benefits from personalization significantly influence their positive attitudes toward the messages [23,27]. Several researchers showed that raising PC due to personalization has restricted consumers from liking the products or services unconditionally [73,76,77]. A similar approach has also been observed in the case of the PAs [69,78]. Consumers' click-through intention toward advertisements has been considered one of the important key performance indicators by several researchers to measure the effectiveness of advertisements [30,79]. Click-through intention shows the motive of the consumers toward the PSMAs. This work has examined the influence of Generation Z consumers’ PU and PC on the intention to click the PAs. The hypothesis developed is mentioned below. The conceptual framework of all the hypotheses has been presented in Fig. 1.

H4 Higher PU creates positive intentions among Generation Z consumers to click the PSMAs.

H5 Higher PC creates negative intentions among Generation Z consumers to click the PSMAs.

## 3 Research design

The explanatory sequential mixed-methods design has been used in this work to validate the conceptual framework (Fig. 1). The framework has been developed by expanding and integrating both the self-congruency theory and the privacy-calculus theory. Covariance-based structural equation modeling technique has been applied in Study 1 to test the hypotheses developed. The details of Study 1 have been mentioned in section 4. After Study 1, it has been observed that the psychological determinants conceptualized under the study have a significant influence on consumers' click-through intentions toward the PAs. In Study 2, in-depth personal interviews were conducted with 15 informants, who also participated in Study 1, to explore the factors associated with consumers’ perceptions, perceived usefulness, and privacy concerns for PSMAs. Three informants from each personality trait have purposively been selected for the qualitative study. The details of Study 2 have been presented under section 5.

**Study population:** Indian individuals, aged between 18 and 27 years, who have social media account(s) on Facebook, and who have used Facebook during past one month from the date when the survey was conducted, have been considered as the study population. India has been considered as the study location because it is a diverse country concerning culture, geography, economic status, as well as religious belief [80]. In India, the number of Facebook users has increased from 190 million users in 2018 to around 441 million users by 2022 [81]. It is more than one-fourth of India's total population.

## 4 Study 1 (quantitative method)

### 4.1 Research design

**Sampling method:** Snowball sampling techniques were used to choose the respondent sample. The digital copy of the questionnaire was prepared in the Google Form and was circulated among the students across Indian universities that offer both undergraduate and postgraduate programs, as well as among the known persons of the researchers. Snowball sampling was adopted to reach a convincing sample size asking the initial respondents either to connect the researchers with their friends or to circulate the questionnaire within their friend circle, who are in the Generation Z age group. The data collection was conducted during February and March 2023. Data was collected from a sample of 336 individuals. After removing the partially filled and non-matching samples’ data, the final respondent sample consists of 324 Facebook users within the age group of 18–27 years. The demographic details of the respondents are provided in Table 1.

**Table 1:** Demographic details of the Respondents.

Demographic Variable	Category	Count (percent)
Gender	Female	124 (38.3 %)
	Male	200 (61.7 %)
Residential Area	Rural	21 (6.5 %)
	Town	149 (46.0 %)
	Capital City	154 (47.5 %)
Time Spent on Social Media in a Day	Less than 1 h	35 (10.8 %)
	1–2 h	122 (37.6 %)
	>2–4 h.	99 (30.5 %)
	More than 4 h	68 (21.0 %)

**Ethical approval:**This study was approved by the Institutional Ethics Committee (Reg. No. – ECR/129/Inst/KL/2013/RR-19) of Amrita Vishwa Vidyapeetham, India (IEC-AIMS-2023-ASB-39) on February 21, 2023. Written informed consent has been taken from all the participants. The study protocol, close-ended questionnaire, and informed consent have been submitted to the Ethics Committee for approval.

**Survey instrument for quantitative method:** A structured questionnaire has been prepared to collect data regarding the sample's personality traits, and general perceptions of Facebook's trait-based PAs. The survey instrument used in the study has seven sections. The first section of the questionnaire describes the study objective in short, discusses the importance of the research, and asks for the consent of the respondents. In the second section, the questions are related to the respondents' personality traits. The questions have been adopted from Costa and McCrae's Big-Five Personality Traits Big Five Inventory (BFI) [2]. Similarly, in the third, fourth, and fifth sections, questions are regarding people's PP, PU, and PC respectively. All the questions are adopted from renowned marketing and advertising research studies [2,27,82]. In the sixth section, the click-through intention of the respondents has been collected. The demographic details of the respondents have been covered under section seven. Other than the seventh section, the responses of other sections are on the five-point Likert Scale where the lowest point is ‘Strongly Disagreed’ and the highest point is ‘Strongly Agree’. The list of constructs, measured variables used under each construct, and the adopted sources have been mentioned in Appendix A.

**Quantitative method:** The Covariance-based Structural Equation Modeling (CB-SEM) technique has been used to check how closely the observed data fit with the conceptual framework diagram mentioned in Fig. 1 [83]. SEM technique has two steps: a. Measurement model and b. Structural model. The measurement model checks the fitness of the model and how constructs have been defined by the measuring variables and the structural model computes the impact of constructs on each other. SPSS AMOS 23 software has been used for analyzing both measurement and structural models and for the hypotheses testing among the constructs.

### 4.2 Results and analysis

#### 4.2.1 Measurement model analysis

**Fig. 2:**
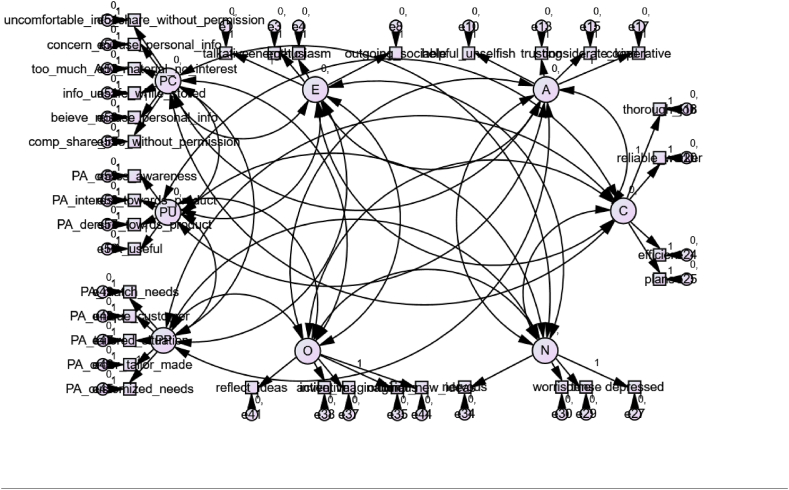
The measurement model with standardized estimates. E: Extraversion, A: Agreeableness, C: Conscientiousness, N: Neuroticism, O: Openness, PP: Perceived Personalization, PU: Perceived Usefulness, PC: Privacy Concerns.

In the first measurement model, Costa and McCrae's 44-item Big Five Inventory (BFI) has been used for defining the personality traits constructs – extraversion, agreeableness, conscientiousness, neuroticism, and openness [26]. Alongside these constructs, five measured variables have been used for the PP construct, four have been used for the PU construct, and five variables have been used for the PC construct. After conducting the measurement model testing, it was found that the factor loadings of a few measured variables under the personality traits constructs are less than 0.5 and also insignificant. After removing those variables, a total of 21 measured variables were used for developing the personality traits construct. The measurement model has been presented in Fig. 2. Factor loadings of all the variables under the remaining three constructs are more than 0.6. The factor loading values of the measured variables under the constructs are mentioned in Table 2 along with the mean and standard deviation values.

**Table 2:** Descriptive Statistics and Factor Loadings of the measured variables on the constructs.

Constructs	Measurement Items	Mean	SD	Factor Loadings
Personality Traits				
A. Extraversion				
	I see myself as someone who is talkative	3.28	1.07	0.472***
	I see myself as someone who is full of energy	3.77	1.01	0.681***
	I see myself as someone who generates a lot of enthusiasm	3.84	0.90	0.705***
	I see myself as someone who is outgoing, sociable	3.57	1.18	0.587***
B. Agreeableness				
	I see myself as someone who is helpful and unselfish with others	4.15	0.89	0.580***
	I see myself as someone who is generally trusting	4.24	0.85	0.624***
	I see myself as someone who is considerate and kind to almost everyone	4.10	0.93	0.677***
	I see myself as someone who likes to cooperate with others	4.17	0.86	0.582***
C. Conscientiousness				
	I see myself as someone who does a thorough job	3.75	1.03	0.596***
	I see myself as someone who is a reliable worker	4.01	0.98	0.566***
	I see myself as someone who does things efficiently	3.90	0.89	0.787***
	I see myself as someone who makes plans and follows through with them	3.35	1.14	0.568***
D. Neuroticism				
	I see myself as someone who is depressed, blue	2.47	1.24	0.484***
	I see myself as someone who can be tense	3.34	1.08	0.680***
	I see myself as someone who worries a lot	3.43	1.16	0.750***
	I see myself as someone who gets nervous easily	3.13	1.18	0.632***
E. Openness				
	I see myself as someone who is original, comes up with new ideas	3.81	0.97	0.704***
	I see myself as someone who is curious about many different things	4.13	0.91	0.535***
	I see myself as someone who has an active imagination	4.13	0.82	0.556***
	I see myself as someone who is inventive	3.48	0.94	0.694***
	I see myself as someone who likes to reflect, play with ideas	3.81	0.90	0.711***
Perceived Personalization				
	PA on Social Network Sites (Facebook) makes purchasing recommendations that match my needs.	3.34	1.16	0.779***
	I think that PA on Social Network Sites (Facebook) enables me to order products that are tailor-made for me.	3.17	1.11	0.781***
	Overall, PA Social Network Sites (Facebook) is tailored to my situation.	3.09	1.09	0.823***
	PA Social Network Sites (Facebook) makes me feel that I am a unique customer.	2.65	1.21	0.625***
	I believe that PA Social Network Sites (Facebook) is customized to my needs.	3.17	1.15	0.747***
Perceived Usefulness				
	I feel that PA is helpful in creating awareness of a product/service	3.90	0.95	0.802***
	I feel that PA is relevant to generate interest towards a product/service.	3.92	0.90	0.810***
	I feel that PA is worthwhile to create a desire towards a product/service.	3.83	1.02	0.776***
	Overall, I feel that PA is useful.	3.78	1.05	0.753***
Privacy Concerns				
	When I receive PA, I feel uncomfortable when information is shared without permission.	3.85	1.14	0.750***
	When I receive PA, I am concerned about misuse of personal information.	3.93	1.10	0.797***
	When I receive PA, it bothers me to receive too much advertising material of no interest.	3.93	1.07	0.626***
	When I receive PA, I am afraid that information may not be safe while stored.	3.88	1.07	0.871***
	When I receive PA, I believe that personal information is often misused.	3.73	1.08	0.822***
	When I receive PA, I think companies share information without permission.	3.86	1.15	0.750***

Notes: * Significant at 5 % level of significance.

** Significant at 1 % level of significance.

***Significant at 0.1 % level of significance.

Several indices have been measured to understand the fitness of the measurement model. The ratio of chi-square to the degrees of freedom of the model is 1.721 which is quite lower than the threshold value of three. The Root Mean Square Error of Approximation (RMSEA) value is 0.047 which is also lower than 0.05 with p value 0.83 which is quite higher than 0.05. The comparative fit index value of the model is quite high (0.91). Parsimonious Normed Fit Index (0.689) and Parsimonious Comparative Fit Index (0.773), both are greater than 0.5. The model satisfies all the standards suggested by Hooper, Coughlan, Mullen [84]and Byrne [85].

##### 4.2.1.1 Constructs validity

Convergent validity and discriminant validity of the measurement model have been reviewed to further measure the accuracy of the theoretical model.

**Factor loadings and statistical significance:** The standardized factor loadings of all the variables have been mentioned in Table 2. All the variables are statistically significant; except one variable of the extraversion construct (0.472) and one of the neuroticism constructs (0.484) have factor loadings less than 0.5. All the other factor loadings are more than 0.5. All the factor loadings are also within −1 to +1 which shows that the variables are not having any problem with the constructs.

**Convergent validity:** The Average Variance Explained (AVE) values of the five constructs related to the personality traits of a consumer, are slightly less than 0.5, whereas the AVE values of all other constructs are more than 0.5. All the Construct Reliability (CR) values are greater than 0.7 [86]. High Construct Reliability values of the constructs explain that the measured variables are consistently representing that particular construct.

**Discriminant validity:** According to Fornell and Larcker [86], discriminant validity can be maintained if the AVE of the construct is greater than the square of the correlations of all the constructs. The suggested benchmark has been maintained by all the constructs. So, the requirements of both convergent validity and discriminant validity have been met by the model. The AVE, CR, and correlation coefficient values are mentioned in Table 3.

**Table 3:** Average Variance Explained (AVE) and Construct Reliability (CR) and Correlation matrix among the constructs.

Constructs	AVE	CR	E	A	C	N	O	PP	PU	PC
Extraversion (E)	0.38	0.71	1							
Agreeableness (A)	0.40	0.72	0.46	1						
Consciousness (C)	0.42	0.74	0.51	0.59	1					
Neuroticism (N)	0.42	0.74	−0.11	0.08	−0.14	1				
Openness (O)	0.43	0.79	0.58	0.46	0.73	−0.06	1			
Perceived Personalization (PP)	0.57	0.87	0.34	0.18	0.28	0.08	0.18	1		
Perceived Usefulness (PU)	0.62	0.87	0.26	0.29	0.23	0.1	0.23	0.62	1	
Privacy Concerns (PC)	0.50	0.88	0.087	0.2	0.14	0.13	0.15	−0.015	0.055	1

#### 4.2.2 Structural model

**Fig. 3:**
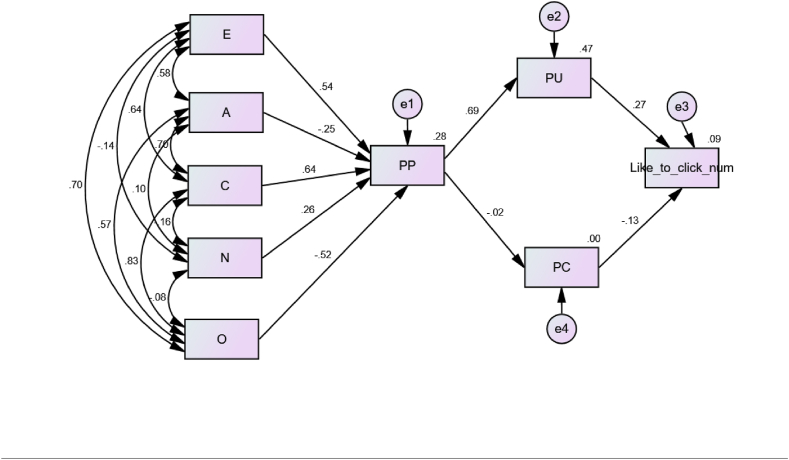
The Structural Model with standardized beta weights. E: Extraversion, A: Agreeableness, C: Conscientiousness, N: Neuroticism, O: Openness, PP: Perceived Personalization, PU: Perceived Usefulness, PC: Privacy Concerns, Like_to_click_num: Click-through intention to PSMAs (0/1).

The structural model along with the standardized beta values have been presented in Fig. 3. All the personality traits have significant relationships with the PP construct. Extraversion (β = 0.54, P < 0.001), conscientiousness (β = 0.64, P < 0.001), and neuroticism (β = 0.26, P < 0.001) have significant positive relationships with PP. Consumers with high scores in extraversion, conscientiousness, and neuroticism personality traits perceive personalization positively. Whereas consumers with high scores in openness (β = −0.52, P < 0.001) and agreeableness (β = −0.26, P < 0.001) perceive personalization negatively. The model also showed that PP has a highly significant strong positive relationship (β = 0.69, P < 0.001) with the PU construct. Yet, PP doesn't have a significant relationship with the PC construct (P value = 0.783). Further, PU has a significant positive influence on the consumers towards their click-through intentions to PSMAs (β = 0.26, P < 0.001) whereas PC has a significant negative influence on the outcome variable (β = −0.13, P < 0.05). All the hypotheses made in the model have been proven true, except that of the relationship between PP and PC.

## 5 Study 2 (qualitative method)

### 5.1 Qualitative research design

After the quantitative analysis, a preplanned in-depth interview was conducted with the help of an interview guide. The interview guide is attached as Appendix B. In-depth interviews are designed to allow researchers to explore the psychological attributes that determine consumers' behaviors toward trait-based PSMAs. An initial sample of 15 informants was selected from the original sample of 324 respondents considered in Study 1 (quantitative study) and researchers were open to interview further informants if new information was still being obtained. These 15 informants were purposively selected in such a way that three informants dominantly represented each personality trait in Study 1. The informants who showed dominant characteristics in more than one personality trait were excluded from the selection for Study 2. The reason for this was to identify if there are distinct factors and patterns of typical personality traits leading to certain type of perceived personalization as was observed in Study 1. Saturation of data was reached at the end of fifteen interviews; hence, no further informant was required to be interviewed. Through these interviews, experiential accounts of informants from each personality type were collected to understand and identify the factors that influence consumers’ perceived personalization, perceived usefulness, and privacy concerns towards trait-based PSMAs.

A thematic analysis was performed on the data thus collected. The thematic analysis followed a six-step process framework: familiarization, coding, generating themes, reviewing themes, defining and naming themes, and writing up [87,88]. Interviews were conducted till data saturation was reached. Open and axial codes were developed through the iterative process. Four crucial themes were identified.

### 5.2 Themes emerged

#### 5.2.1 Perceived personalization

Theme1 Personalized ads associated with previous searches by self, family, and friends

One of the crucial themes that we found under perceived personalization states that PAs are associated with previous searches, not only by the consumers themselves but also by their family members, friends, and acquaintances. This perceived meaning of PA by informants is similar to its existing theoretical definition. Informants perceived these ads as push ads. Furthermore, it was found that among the five personality types, extroverts, conscientious, and neurotic personalities feel these personalized ads are based on previous searches and therefore are helpful in prescribing better search options and a variety of products. Personalities that are predominantly agreeable or open to experience, though perceive these ads as providing variety, they feel too much information is shared with platforms and therefore have trust issues with such ads.

While extroverts show a personality type where they like to share information, would prefer to take assistance from such personalization, and use the data on products that they are supplied with by the platforms. Conscientious personalities prefer time-saving techniques to increase efficiency therefore rather than being wary of too much information sharing in the portals, they appreciate these personalized ads for saving time to search for new options of products and services.

Since neurotic personalities desire to stay relevant among people, they prefer personalized ads, as these ads provide them a chance to share updated information about products with their relevant groups.

While few personality types appreciate and benefit from these personalized ads and perceive them as useful, personality types such as agreeable ones do not prefer these ads. Despite the benefits that these ads may provide of reducing search time and effort, agreeable people feel unsure of the platforms where they have to reveal too much personal information and thereby may end up in confrontation or risk of personal data misuse by such platforms. Similarly, people who are open-to-experience feel a lack of thrill or excitement in the personalized ads due to repetitive and monotonous suggestions provided in these personalized ads.

As mentioned by an informant:

‘Based on my previous online shopping experience and what keywords I've searched, on the basis of that new advertisements were displayed.’

#### 5.2.2 Perceived usefulness

Theme 2 Personalized advertisements are found more helpful than general advertisements by improving consumers' buying decision process

Under perceived usefulness, informants mentioned that PAs are found to be more helpful than general advertisements. These advertisements benefit consumers by improving their buying decision process by providing recommendations for more products in the same category and allied categories. Such recommendations not only ease the process of search but also reduce the time required for search.

As mentioned by an informant:

‘But at other times when I am searching for something and I end up getting something related to it and it's more personalized, more customized, then it is really nice because I don't have to put more effort. I don't have to go more in-depth into finding.’

Similarly, one of the informants also mentioned that PAs not only provide us with a variety of options in products but also provide the best prices available.

#### 5.2.3 Privacy concerns

Theme 3 Concern regarding usage of personal data

Informants raised concerns regarding the usage of personal data for the development of PAs as well as sharing those ads through different social media platforms. However, the level of concern varies across different personalities.

As mentioned by an informant:

‘I have privacy concerns since my data would be shared with a lot of people, websites, and platforms’.

Informants are comfortable sharing their personal information with trusted organizations. They also raised concerns that organizations are sharing their personal data with other parties without their consent.

Informant mentioned:

‘Yes, for some years there are trusted apps that I actually know and those are the same apps coming in my Instagram or Snapchat.’

They mentioned that if the communication content is highly personalized and shared on all the platforms repeatedly, it raises severe privacy concerns among the consumers.

Informant:

‘I would say that the level of personalization. As I mentioned, it shouldn't be at the level where you know so much about consumers.’

Informants also observed that advertisements of related products and services are displayed on their social media platforms about which they have spoken with someone either over the phone or physically, while the mobile phone was kept nearby. The informants believe that their voices get captured and used for personalized product promotions.

As mentioned by an informant:

‘There are times when I'm talking about something and suddenly some ads pop up even though I haven't searched for it anywhere. But I have spoken about it when I'm closer to my phone and then some ads end up coming automatically in my social media.’

Some of the informants showed concerns about consumers' knowledge of actual digital advertisements and fake advertisements which may lead them to harmful websites.

As mentioned by an informant:

‘Consumers should be educated enough to know that it is a scam ad or it might land on to a different landing page.’

#### 5.2.4 Click-through motivations

Theme 4 Privacy paradox & more

Informants mentioned that they click PSMAs after reviewing the advertisements thoroughly such as which platforms the advertisements have been shared, whether the information shared is relevant or not, whether the advertisement is original and not fake, etc. Consumers carefully assess both the benefits and the risks associated with PA before clicking them.

As mentioned by an informant:

‘I go through it, then I go to their page and check the like number of followers or the reviews they get.’

The influence of customers’ state of mind on the click-through intention of the advertisement was also emphasized by the informants. If the consumer is not engaged in some work during the display of the advertisement or is in a happy mood, there is a higher chance that the person will click on the advertisement.

As mentioned by an informant:

‘Maybe at one point in time, you might get him in the right mood or him or her in the right circumstances that you're pitching the advertisement.’

One of the crucial codes that came up regarding click-through motivation is the ‘trustworthiness of the Ad sharing digital platform’. Informants are comfortable clicking the personalized ads displayed on their preferred and trusted platforms, but they refrain from engaging with similar ads displayed on unknown websites. A few informants prefer to visit the original website or e-commerce platform directly rather than clicking the personalized social media advertisements if they prefer the product or services.

As mentioned by an informant:

‘Most of the cases what happened is I will go to that particular trusted website or that application and then I'll filter out my choice.’

## 6 Discussion

The main objective of this study is to understand how Generation Z consumers with different personality traits perceive the PSMAs, how their PP influences PU and PC associated with those advertisements, and finally show the intention to click on PSMAs. The findings of Study 1 (quantitative analysis) show that Generation Z consumers with different personality traits perceive PSMAs very differently. Consumers' PP positively influences the PU of the PSMAs, but it does not have any significant influence on the PC of the consumers. It also finds that PU has a positive influence and PC has a negative influence on consumers’ click-through intentions of the PSMAs.

Extant literature attempting to test the self-congruity theory verified that consumers' self-congruity is an important factor for the success of PAs [5,17,25]. This study has also supported the phenomena. The analysis extended the theory by confirming that Generation Z consumers with different dominant personality traits perceive personalized communications differently and subsequently act on those differently. Further privacy calculus theory has been extended in this study to understand the influence of consumers' PP on their personalization-privacy paradox. The findings revealed that Generation Z consumers' high PP towards PSMAs increases the PU of communications and subsequently increases the click-through intentions, which is supported by a body of earlier research [5,70]. Freya De Keyzer and team [5] confirmed in their work that consumers' PP has a negative impact on consumers' perceived intrusiveness. However, in this study, it has been observed that the PP of Generation Z consumers has no impact on their PC related to PSMAs. Further, the relationships between consumers’ PU and PC and click-through intentions towards PSMAs found in this study, are similar to the previous studies [5,23,30].

In Study 2, in-depth interviews were conducted to explore the potential causes of those relationships observed in Study 1. All the in-depth interview participants mentioned that they perceived PSMAs on Facebook as personalized because of their associations with the previous searches conducted by themselves, family, friends, and acquaintances. Study 1 confirmed that extraversion and conscientiousness dominant consumers' PP as highly positive. During in-depth interviews, extroverted consumers confirmed that PSMAs enhance their knowledge and subsequently their prestige among their connections. The result supports the previous finding that extroverted consumers love to get connected with people, society, and nature. PSMAs help them to gather new knowledge that they can share with others [35]. Individuals with conscientiousness trait do not want to waste time and PSMAs appear useful for them in reducing their search efforts and save time on the Internet. A similar insight has been portrayed in another research work conducted by Dupre et al. on job performance [89]. Neurotic consumers also positively perceive personalization but not as high as extraversion and conscientiousness traits. In the personal interviews, neurotic informants mentioned that by sharing personalized ads in their social circles they keep themselves relevant and emotionally connected. Buss and Plomin [90] mentioned that neurotic consumers showed a strong association with emotional content.

According to the quantitative model, consumers with openness to experience and agreeableness personality traits carry negative perceptions towards the PSMAs. Openness to experience dominant consumers prefer generic communications to personalized ones so that they can explore new communications rather than something very personalized whereas consumers with agreeableness characteristics are concerned about usage of personal data. Sun and Wu [91]mentioned in their work that agreeable dominant people show excessive control toward their usage of social media to avoid any unnecessary conflict.

Study 1 confirmed that consumers with high PP have considered PSMAs to be more useful. The qualitative study explored that consumers find PSMAs useful because they help consumers to make informed decisions by providing suitable recommendations of required as well as allied products and services. Previous literature has observed that PP has a positive impact on consumers' perceived relevance toward PSMAs [5,92]. However, study 1 also showed that PP does not have any influence on the PC of the consumers. Study 2 revealed that all consumers irrespective of personality traits have PC towards PSMAs since consumers’ personal information is used to develop those ads. Consumers' sense of less control over their personal information develops their PC [27].

Previous literature has shown the impact of the personalization-privacy paradox concept on the consumers’ engagement with the PAs [[93], [94], [95]]. The dynamics between the PU and the PC play a crucial role in the effectiveness of the PAs. Like the previous studies, in Study 1 it has been observed that PU has a positive likelihood to click the PSMAs whereas the PC acts negatively on the same [27,75]. Consumers mentioned during in-depth interviews that the state of mind of the consumer at the time of seeing the PSMAs and the trustworthiness of the ad-sharing platform influence the click-through intentions of the consumers. The data scandal cases with Facebook have broken consumers' trust on the platform [96].

**Managerial contribution:** Overall findings of this study help business managers by providing them with a sketch of underlying dynamics among personality traits, PP, PU, and PC, that lead to engagement with the PSMAs. PP, PU, and PC, and finally the click-through intention varies across personality traits, and personalization should be crafted accordingly for better customer engagement. However, even though personalized communications have more persuasive capability than generic ads; it has also been observed that personalized communications can also be a reason for irritation among consumers, which subsequently can destroy the brand image among prospective buyers. The knowledge generated by this study suggests companies to prepare more focused and strategic communications for consumers based on their personality traits. With the easy availability of ‘digital footprints’ of consumers and the advent of new technologies, companies can easily prepare consumers' characteristics profiles as well. At the same time, too detailed personalization based on personal trait analysis may incite privacy concerns further, even among those who would have otherwise been more open to personalization. There have been inconsistent observations while mapping personality traits with consumers' attitudes toward products [17]. Hence, companies should assess the level of acceptability among different customers for different products. Since the pattern of perceived usefulness and privacy concern vary for individuals with different characteristics, as well as may also vary for different products within the same personality type, this study recommends advertisers use Artificial Intelligence to correctly capture the personality types and design the social media advertisements accordingly [97]. Personalization-privacy paradox may decline consumer engagement if not strategically designed [98]. Not using publicly available information to woo potential customers by customizing advertisements for them is foolishness. However, using personal information more than necessary is not wise and can be disastrous for a brand.

## 7 Limitations and future research

The study offers a rare analysis of the association between consumers’ personality traits and PP. Further efforts to understand the pattern of this association across different socio-demographic groups of population and geographies will help the researchers and marketers to create a generalized understanding, aiding them towards more effective decision-making. Generation Z consumers grew up in a digitized ecosystem, and therefore they are less worried in terms of their presence in online platforms. Yet, the lack of a distinct association between PP and PC solicits more studies to explore the possible reasons and contexts for such pervasive PC among Generation Z consumers. Since the central focus of this study is to understand the impact of Perceived Personalization on click-through intention on PSMAs through the assessment of the impact of privacy paradox i.e. the trade-off between PU and PC, the analysis has not examined the mediation effect of PU and PC individually on intention to click. The influence of PU and PC on click-through intentions is a strong scope of future research.

This cross-sectional study provides a broad understanding of how several factors impact the click-through intentions toward PSMAs among Indian Generation Z consumers with different personality traits. We acknowledge the fact that since this study has not adopted any experimental approach, it could not examine content-specific experiments on the acceptability of PSMAs. Future efforts can be made to assess how the PP and privacy paradox of Generation Z consumers varies based on the contents of the advertisements and across the brands or types of products (such as hedonic and utilitarian products). However, researchers have shown that the effectiveness of personalized messages is comparatively much higher over non-personalized messages when there is a demand for the cognitive association of the consumer [99], this study could not expand its horizon to examine the variations associated with cognitive loads. Further research conducted among the Generation Z population in such directions will be impactful. Because the majority of our sample was at least enrolled in an undergraduate program and were residents of urban locations during the time of the survey, the other limitation that can be stated is our findings may not be generalizable across all sections of the Generation Z population. Nevertheless, this does not narrow down the practicality of our research, since the choice of products that are available on social media is mostly limited in urban areas. Future studies can assess the studied associations for PAs circulated in other relevant online platforms. Privacy policy regulations should also be studied, and appropriate measures should be implemented so that PAs through social media earn more acceptability among consumers.

## 8 Conclusion

This study has demonstrated the varying impacts of personality traits of Generation Z consumers on their PP of PSMAs. Even though positive PP leads to higher PU, PC is omnipresent. Privacy paradox significantly influences click-through intentions. Results confirmed that Generation Z consumers' perceptions toward PAs vary along with the personality traits of the consumers. This knowledge is a noble addition to the field of self-congruency theory. The observation that consumers' PP has a significant influence on consumers’ personalization-privacy paradox which subsequently impacts consumers' digital advertisement click intentions meaningfully contributes to the empirical understanding of the privacy calculus theory.

The results of this research have not only extended the existing theories but also show significant relevance for digital marketing managers. These findings suggest that marketers must try to assess the consumers’ personality traits while designing and circulating PSMAs to the consumers. While developing a PSMA, the advertising experts should keep in mind that extrovert, conscientiousness, and neurotic demonstrate a positive association with perceived personalization, while they should avoid strong personalization for consumers with dominant openness to experience and agreeableness traits.

The findings of this study collectively point towards the larger need to address the cognitive capacity of the consumers through the contents of the advertisements, while creating perceived usefulness. Next, since privacy concerns are almost ubiquitous in the context of PSMAs, marketers should focus on creating perceived privacy protection among consumers. Additionally, both academic and managerial interests should investigate the intrusiveness of personalized advertisements in terms of timing, frequency, and channels.

## Data availability statement

Data will be made available on request.

## CRediT authorship contribution statement

**Partha Saha:** Writing – review & editing, Writing – original draft, Supervision, Project administration, Methodology, Formal analysis, Data curation, Conceptualization. **Angan Sengupta:** Supervision, Formal analysis, Conceptualization. **Priya Gupta:** Writing – review & editing, Writing – original draft, Methodology.

## Declaration of competing interest

The authors declare that they have no known competing financial interests or personal relationships that could have appeared to influence the work reported in this paper.
