# Supplementary material for: Influence of personality traits on generation Z consumers' click-through intentions towards personalized advertisements: A mixed-methods study
Source: Heliyon. 2024 Jul 17;10(15):e34559. doi: 10.1016/j.heliyon.2024.e34559 (PMC11320156; doi:10.1016/j.heliyon.2024.e34559)
Supplement: Multimedia component 1 [file mmc1.docx]

**Appendix A: Constructs and Measurement Items**

| Constructs | Source | Measurement Items |
| --- | --- | --- |
| Personality Traits | Adopted from  (Costa & McCrae, 1992) |  |
| Extraversion (E) |  |  |
|  |  | I see myself as someone who is talkative |
|  |  | I see myself as someone who is full of energy |
|  |  | I see myself as someone who generates a lot of enthusiasm |
|  |  | I see myself as someone who is outgoing, sociable |
| Agreeableness (A) |  |  |
|  |  | I see myself as someone who is helpful and unselfish with others |
|  |  | I see myself as someone who is generally trusting |
|  |  | I see myself as someone who is considerate and kind to almost everyone |
|  |  | I see myself as someone who likes to cooperate with others |
| Conscientiousness (C) |  |  |
|  |  | I see myself as someone who does a thorough job |
|  |  | I see myself as someone who is a reliable worker |
|  |  | I see myself as someone who does things efficiently |
|  |  | I see myself as someone who makes plans and follows through with them |
| Neuroticism (N) |  |  |
|  |  | I see myself as someone who is depressed, blue |
|  |  | I see myself as someone who can be tense |
|  |  | I see myself as someone who worries a lot |
|  |  | I see myself as someone who gets nervous easily |
| Openness (O) |  |  |
|  |  | I see myself as someone who is original, comes up with new ideas |
|  |  | I see myself as someone who is curious about many different things |
|  |  | I see myself as someone who has an active imagination |
|  |  | I see myself as someone who is inventive |
|  |  | I see myself as someone who likes to reflect, play with ideas |
| Perceived Personalization (PP) | Adopted from  (Baek & Morimoto, 2012) |  |
|  |  | Personalized advertising on Social Network Sites (Facebook) makes purchasing recommendations that match my needs. |
|  |  | I think that personalized advertising on Social Network Sites (Facebook) enables me to order products that are tailor-made for me. |
|  |  | Overall, personalized advertising Social Network Sites (Facebook) is tailored to my situation. |
|  |  | Personalized advertising Social Network Sites (Facebook) makes me feel that I am a unique customer. |
|  |  | I believe that personalized advertising Social Network Sites (Facebook) is customized to my needs. |
| Perceived Usefulness (PU) | Adopted from  (Gironda & Korgaonkar, 2018) |  |
|  |  | I feel that personalized advertising is helpful in creating awareness of a product/service |
|  |  | I feel that personalized advertising is relevant to generate interest towards a product/service. |
|  |  | I feel that personalized advertising is worthwhile to create a desire towards a product/service. |
|  |  | Overall, I feel that personalized advertising is useful. |
| Privacy Concerns (PC) | Adopted from  (Baek & Morimoto, 2012) |  |
|  |  | When I receive personalized advertising, I feel uncomfortable when information is shared without permission. |
|  |  | When I receive personalized advertising, I am concerned about misuse of personal information. |
|  |  | When I receive personalized advertising, it bothers me to receive too much advertising material of no interest. |
|  |  | When I receive personalized advertising, I am afraid that information may not be safe while stored. |
|  |  | When I receive personalized advertising, I believe that personal information is often misused. |
|  |  | When I receive personalized advertising, I think companies share information without permission. |
