# Supplementary material for: Influence of personality traits on generation Z consumers' click-through intentions towards personalized advertisements: A mixed-methods study
Source: Heliyon. 2024 Jul 17;10(15):e34559. doi: 10.1016/j.heliyon.2024.e34559 (PMC11320156; doi:10.1016/j.heliyon.2024.e34559)
Supplement: Multimedia component 2 [file mmc2.docx]

**QUALITATIVE INTERVIEW SCHEDULE**

1. Have you ever come across any personalized social media advertisements?
2. Have you ever clicked or shown interest in clicking those advertisements on any social media platform?
3. If yes, why? If no, why?
4. In your opinion, which type of products or services are suitable for personalized ads through social media and why?
5. How did you feel about those personalized advertisements you saw on social media?
6. In which ways those personalized advertisements can be useful for you?
7. Is there any concern related to privacy? If yes, why and of what kind? If no, what are the reasons that you find online personalized advertisements are safe?
8. What factors in a personalized advertisement motivate you to click through those websites or channels? OR

How do you get motivated to click through the websites or channels of a personalized advertisement?

1. How do you define your personality? Do you think that personalized advertisements connect with /go well with your personality? If yes, why and if no, why?
